# Catch composition and life history characteristics of sharks and rays (Elasmobranchii) landed in the Andaman and Nicobar Islands, India

**DOI:** 10.1371/journal.pone.0231069

**Published:** 2020-10-29

**Authors:** Zoya Tyabji, Tanmay Wagh, Vardhan Patankar, Rima W. Jabado, Dipani Sutaria

**Affiliations:** 1 Andaman Nicobar Environment Team, North Wandoor, South Andaman, Andaman and Nicobar Island, India; 2 Wildlife Conservation Society, Kodigehalli, Bengaluru, Karnataka, India; 3 Elasmo Project, Dubai, United Arab Emirates; 4 College of Science and Engineering, James Cook University, Townsville, Australia; Consejo Nacional de Investigaciones Cientificas y Tecnicas (CONICET), ARGENTINA

## Abstract

Detailed information on shark and ray fisheries in the Andaman and Nicobar Islands, India are limited, including information on the diversity and biological characteristics of these species. We carried out fish landing surveys in South Andamans from January 2017 to May 2018, a comprehensive and cost-effective way to fill this data gap. We sampled 5,742 individuals representing 57 shark and ray species landed from six types of fishing gears. Of the 36 species of sharks and 21 species of rays landed, six species of sharks (*Loxodon macrorhinus*, *Carcharhinus amblyrhynchos*, *Sphyrna lewini*, *C*. *albimarginatus*, *C*. *brevipinna*, and *Paragaleus randalli)* comprised 83.35% of shark landings, while three species of rays (*Pateobatis jenkinsii*, *Himantura leoparda* and *H*. *tutul*) comprised 48.82% of ray landings, suggesting a species dominance in the catch or fishing region. We provide insights into the biology of species with extensions in maximum size for seven shark species. Additionally, we document an increase in the known ray diversity for the islands and for India with three previously unreported ray species. We found that amongst sharks, mature individuals of small-bodied species (63.48% males of total landings of species less than 1.5 m total length when mature) and immature individuals of larger species (84.79% males of total landings of species larger than 1.5 m total length when mature) were mostly landed; whereas for rays, mature individuals were predominantly landed (80.71% males of total landings) likely reflecting differences in habitat preferences along life-history stages across species and fishing gear. The largest size range in sharks was recorded in landings from pelagic longlines and gillnets. Further, the study emphasizes the overlap between critical habitats and fishing grounds, where immature sharks and gravid females were landed in large quantities which might be unsustainable in the long-term. Landings were female-biased in *C*. *amblyrhynchos*, *S*. *lewini* and *P*. *jenkinsii*, and male-biased in *L*. *macrorhinus* and *H*. *leoparda*, indicating either spatio-temporal or gear-specific sexual segregation in these species. Understanding seasonal and biological variability in the shark and ray landings over a longer study period across different fisheries will inform future conservation and fishery management measures for these species in the Andaman and Nicobar Islands.

## Introduction

Elasmobranchs (sharks and rays) are recognized as one of the marine taxa with the highest extinction risk and in need of urgent conservation measures [1]. Despite considerable inter-specific and intra-specific life-history variation [2, 3], most species have relatively low productivity making them highly susceptible to anthropogenic and natural stressors [4]. Populations of many species have drastically declined globally due to overfishing and habitat degradation, raising concerns about their long-term survival [1].

In the past few decades, India has consistently been one of the top three shark and ray harvesters in the world, contributing an average of 67,391 metric tonnes of sharks, rays, and chimaeras annually between 2007 and 2017 [5, 6]. Here, sharks and rays are primarily caught as bycatch [7–11] in a large fishing fleet of 269,047 registered commercial and artisanal fishing vessels [12] targeting a range of commercially important pelagic and demersal species. However, a few targeted shark fisheries that formed in the 1980s remain active, including in the Andaman and Nicobar Islands [13, 14].

The Andaman and Nicobar Islands account for <1% of the total marine fish landings of the country, despite the Exclusive Economic Zone of the islands representing 29.7% of that of India [15]. The fishing sector forms an important source of livelihood, as well as providing food security and employment, and is considered to be a major resource for the economy of the islands. Fish from the family Lutjanidae, Carangidae, Thunninae, Engraulidae, and Clupeidae constitute the majority of landings [16]. Currently, most fish resources are considered under-exploited with an estimated average of 30,000 tonnes fished during the year 2007–2008 [15], accounting for 19% of the fishery potential [15, 17]. However, fisheries show declining trends of the mean trophic level of catches [18, 19].

The marine fish catch reconstruction for the islands indicates that an estimated 60,000 tonnes of sharks and rays were landed from commercial and artisanal fisheries in 2010 [19]. Anecdotal information from interviews with fishers on these islands indicate that shark and ray populations have declined [20], but there have been limited surveys of landings carried out to assess the current situation. This limited information on species and stocks may have detrimental effects, not only on the ecology of these animals, but also on the sustainability of these fisheries and the food security they provide, as well as on the socio-economic dependence of fisher communities [21, 22].

Over the years, with growing reports of declining populations of sharks and rays, the Government of India has implemented several conservation policies. In 2001, ten species of sharks and rays, including the whale shark *Rhincodon typus*, knifetooth sawfish *Anoxypristis cuspidata*, Pondicherry shark *Carcharhinus hemiodon*, Gangetic shark *Glyphis gangeticus*, speartooth shark *G*. *glyphis*, Ganges stingray *Himantura fluviatilis*, freshwater sawfish *Pristis microdon* (*= P*. *pristis*), green sawfish *P*. *zijsron*, giant guitarfish *Rhynchobatus djiddensis* (see discussion), and porcupine ray *Urogymnus asperrimus* were listed under Schedule I of the Indian Wildlife (Protection) Act, 1972 (WLPA). In 2009, the Andaman and Nicobar Islands Fisheries Regulation declared a 45-day closed season for shark fishing from April 15^th^ to May 31^st^ around the islands through the prohibition of shark and tuna pelagic longlines and trawl nets. In 2013, the Ministry of Environment, Forest and Climate Change declared a ‘Fin-attached Policy’ where sharks have to be landed whole, with their fins naturally attached to their bodies. In 2015, India’s Ministry of Commerce and Industry issued a notification prohibiting the export of all shark fins. While these management policies, if properly implemented, are a positive step for shark conservation in India, there appears to be an agenda mismatch between the Ministry of Environment, Forest and Climate Change and the Ministry of Animal Husbandry, Dairying and Fisheries, with the latter having recently developed a strategy to expand fisheries and increase yield. This expansion includes developing new schemes and projects to harness fishing potential and create employment opportunities, by issuing additional fishing licenses, building infrastructure such as cold storage centers, blast freezers and ice plants, and increasing introduction of deep-sea, motorized and mechanized boats [23].

In order to develop best management practices, basic life-history information such as age, growth, and maturity is required to form the basis of population assessments. However, in many developing countries, including India, landings remain unmonitored and unregulated with little species-specific data collected, which hampers population assessments [22]. Additionally, since different species can exhibit geographic variability in biological traits, such as size at birth, size at maturity, maximum size, litter size, and breeding cycle [24–26], it is important to undertake region-specific studies so they can inform local management strategies. Most literature on sharks and rays from the Andaman and Nicobar Islands has been limited to species identification and taxonomy [27–32]. A large knowledge gap exists in our understanding of the catch composition of commercial species landed, their population trends and biological characteristics across seasons. Here, we aim to address this gap by assessing sharks and rays landed in the Andaman and Nicobar Islands and exploring 1) the species composition across seasons; 2) biological information, including size frequency, sex ratio, maturity and length-weight relationships; and 3) characteristics of fishing gears and grounds where sharks and rays were reportedly fished.

## Materials and methods

### Study area

**Fig 1:**
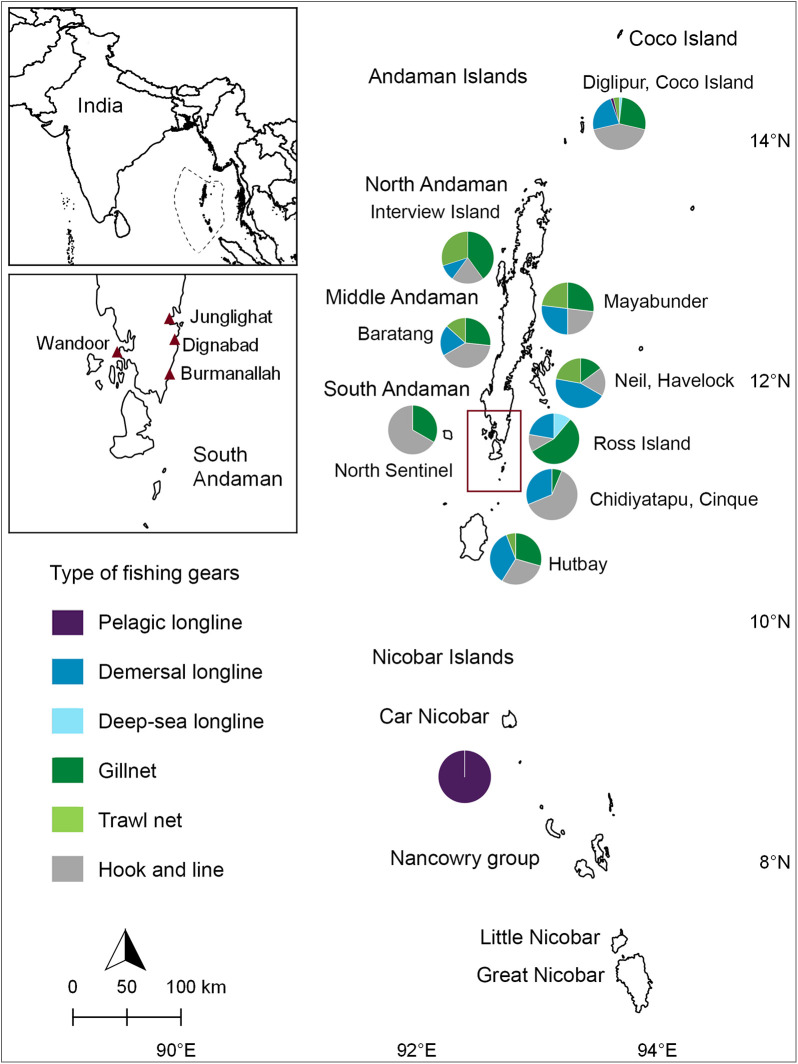
Map of the sampling sites and fishing grounds of the Andaman and Nicobar Islands, India. Top left: Map of India with the Exclusive Economic Zone boundaries of Andaman and Nicobar Islands demarcated; Bottom left: Map of South Andaman with maroon triangles indicating sampled fish landing centers; Right: Map of the Andaman and Nicobar Islands showing fishing gear utilization across fishing grounds around the islands. South Andaman is demarcated by the maroon inset. The figure was produced using QGIS Development Team 2019. QGIS Geographic Information System. Open Source Geospatial Foundation Project (http://qgis.osgeo.org).

The Andaman and Nicobar Islands (6°N–14°N and 92°E–94°E) are located in the Bay of Bengal and constitute 29.7% of the total Exclusive Economic Zone of India (Fig 1), covering a coastline of 1,962 km (contributing to 26.10% of India’s coastline). Being oceanic islands, the continental shelf area along these islands is limited in extent, but totals about 16,000 km^2^, with an almost absent continental slope. [23]. The islands experience heavy monsoon from the end of May to September when the south-west monsoon sets in, as well as intermittent or light to heavy rainfall when the north-east monsoon starts in November. For the duration of our study, we characterized landings according to the following seasons: north-east monsoon (NE) (October–January), inter-monsoon or dry season (DS) (February–May), and south-west monsoon (SW) (June–September).

A total of 2,784 fishing vessels are currently active with 7,034 licensed fishers [23]. Sharks and rays are targeted using pelagic and deep-sea longlines, and are caught as bycatch using demersal longlines, trawl nets, gillnets, and hook and line. Vessels with engines of more than 30 horsepower are permitted to fish exclusively beyond six nautical miles and up to 12 nautical miles from the coast (i.e. pelagic longliners and trawlers). In contrast, vessels fitted with engines of 30 horsepower or less and non-mechanised boats are permitted to fish from the high tide up to six nautical miles (i.e. demersal longliners, hook and line, and gillnet fisheries, which operate near the coast and shallow seamounts). Fishers from the Andaman Islands fish across the waters of the Andaman and Nicobar Islands while the communities on the Nicobar Islands, due to their seclusion, only fish for subsistence or to sell fish in the local market [32, 33].

South Andaman is recognized as the most active fishing center in the islands, alone accounting for 74% of the total marine fishing landings [15]. Exploratory visits to landing sites in 2016 across the South Andaman Islands revealed that the majority of sharks and rays fished throughout the Andaman and Nicobar Islands are landed at Junglighat (Fig 1). Junglighat, located in Port Blair, the main city of the Andaman Islands, is the largest fish landing center of the islands with proximity to storage centers and export facilities (Fig 1). We therefore focused our sampling at this location. However, opportunistic surveys were also undertaken at the fish landing sites of Burmanallah, Wandoor, and Dignabad (Fig 1) when fishers or informants reported landings of sharks and rays to the survey team.

### Sampling effort

Fish landing surveys were undertaken from January 2017 to May 2018 for sharks and from October 2017 to May 2018 for rays. Junglighat was visited every alternate day or when the weather permitted from 0600 to 1000 hrs, whereas the remaining site visits were dependent on reports by the informants. As the pelagic longliners from Junglighat directly offload and transport their landings to the processing and storage units, sampling of landings from these vessels was conducted at these units between 1000 to 1400 hrs.

Sharks and rays landed were identified to the species level using the available literature and photo-documented [34–37]. Rays were often landed with their tails cut, in piles, and, in a few cases, when landings were large, accurate pictures and/or measurements were not possible. Therefore, species which were difficult to differentiate morphologically, such as *Neotrygon* sp. and *Pastinachus* sp., were grouped at the genus level and have therefore been excluded from the analysis of the full data set.

For sharks, guitarfishes, and wedgefishes, the total length (TL, a straight line from the tip of the snout to the tip of the tail, with tail flexed down to midline) was measured, whereas for rays, the disc width (DW, a straight line at the widest region of the disc) was measured to the nearest millimeter [35, 36]. Sex was determined by the presence of claspers indicative of males or the absence of claspers indicative of females. For males, the degree of calcification and length of claspers determined the maturity levels. This was categorized from 1 to 3 where (1) refers to immature individuals whose claspers were non-calcified and pliable, and whose length was less than the pelvic fins, (2) refers to maturing individuals whose claspers were partially calcified and semi-pliable, and whose length was longer than the pelvic fins, and (3) refers to fully mature individuals whose claspers were fully calcified and non-pliable [38]. Gravid females were recorded by the presence of emerging embryos or if these could be clearly observed by pressing the abdomen. Whenever possible, gravid females were dissected to record the sex and size of embryos. Young-of-the-year (YOY) individuals were identified by the presence of open umbilical scars which usually close after the first few months of life [38]. Closed or faint scars may sometimes be visible for many months after life, with the amount of time varying between species. Thus, individuals having partially closed or shut but visible umbilical scars have not been included in this study.

Weights were recorded to the nearest gram using a hand-held digital weighing balance for smaller individuals or, whenever possible, weights were provided by the fishers using a circular weighing balance for larger individuals (> 50 kg).

For each boat that landings were sampled from, fishers were approached to gather and record information on the fishing gears used to catch the sharks and rays as well as the fishing grounds.

### Data analysis

Patterns in species, sex, and sizes caught across various seasons and gears were produced using the Python libraries, matplotlib and seaborn [39, 40]. Tentative fishing grounds, including usage of fishing gears, were produced using QGIS Development Team 2019. QGIS Geographic Information System. Open Source Geospatial Foundation Project (http://qgis.osgeo.org).

The hypothesis of equal sex ratios for species where ≥ 50 individuals were sampled, was tested using Chi-square where significance was considered at p < 0.05 [41]. The hypothesis of shark TL being equally caught across different fishing gears was tested using one-way analysis of variance, where significance was considered at p < 0.05 [41].

For sharks, species where > 150 individuals were sampled, size-class frequency distributions by sex and seasons were plotted. The size at 50% maturity (TL_50_) for males was calculated. This was done by fitting the following logistic function to the proportion of mature individuals in 10 or 20 cm size categories: P = 1 / (1 + exp (- r (TL_mid_—TL_50_))), where P is the proportion of mature fish in each length class, TL_mid_ is the midpoint of the length class, TL_50_ is the mean size at sexual maturity, and r is a constant that increases in value with the steepness of the maturation schedule.

Finally, length-weight relationships were determined using regression analysis. The equation W = aL^b^ was converted into a linear form In (W) = In (a) + b In (L), where W is the weight in grams, L is the length in centimeter, ln(a) is the intercept and (b) the slope or regression coefficient. Gravid females were excluded from this analysis as they would skew the length-weight relationship for the species.

### Ethics statement

All individuals examined in this study were landed at the fish landing sites and were already dead upon inspection. No permits or ethical statements were required for the described study, which complied with all relevant regulations.

## Results

Sampling was conducted on 216 days with landings recorded from 567 boats and a total of 5,742 sharks and rays representing 57 species. Of these, 4,632 individuals represented 36 shark species from 18 genera and 11 families while 1,110 individuals represented 21 ray species, 14 genera and eight families. The next section first provides an overview of the information collected on sharks and then rays separately including species composition, species susceptibility to fishing gear, and biological data of the most caught species.

### Shark species composition

Species from the Carcharhinidae family dominated landings and accounted for 19 of the 36 species (82.98% percentage by number). The six most dominant shark species landed were *Loxodon macrorhinus* (n = 1,549, 33.44%), *Carcharhinus amblyrhynchos* (n = 1,215, 26.23%), *Sphyrna lewini* (n = 421, 9.09%), *C*. *albimarginatus* (n = 295, 6.36%), *C*. *brevipinna* (n = 212, 4.57%), and *Paragaleus randalii* (n = 169, 3.64%), constituting 83.35% (percentage by number) of all landings. A species list and summary of biological data for shark is provided in Table 1.

**Table 1:** Summary of biological data for sharks landed.

Species	n	n by sex of individuals	Size (TL cm)	Additional notes
SQUALIFORMES				
Squalidae				
*Squalus hemipinnis*	1	F: 1	F: 66	Specimen caught using hook and line.
Centrophoridae				
*Centrophorus atromarginatus*	1	M: 1	M: 72.5	Specimen caught using deep-sea longline.
*Centrophorus granulosus*	6	F: 3, M: 3	F: 93.5–103 (97 ± 5.22)	All specimens caught using deep-sea longline.
			M: 82.5–92.5 (87.66 ± 7.68)	
ORECTOLOBIFORMES				
Hemiscyllidae				
*Chiloscyllium griseum*	2	F: 1, M: 1	F: 86, M: 84	Both specimens caught using hook and line.
*Chiloscyllium hasseltii*	1	F: 1	F: 88	Specimen caught using trawl net.
Ginglymostomatidae				
*Nebrius ferrugineus*	8	F: 3, M: 4, UK: 1	F: 198–273.5 (247.5 ± 42.88)	Seven of the eight individuals landed in February and March 2017 and 2018. Size extension by 47.2 cm recorded.
			M: 175.9–367.2 (261.15 ± 59.10)	
LAMNIFORMES				
Odontaspididae				
*Carcharias taurus*	1	F: 1	F: 129.4	
Alopiidae				
*Alopias pelagicus*	28	F: 11, M: 9, UK: 8	F: 131.4–272.6 (224.2 ± 66.96)	Twenty-two of the 28 specimens caught in February. All specimens caught in pelagic longlines.
			M: 138.8–270.5 (215.76 ± 59.67)	
*Alopias superciliosus*	6	F: 3, M: 3	F: 210–306.5 (258.25 ± 36.12)	Four of the six specimens caught in February. All specimens caught in pelagic longlines.
			M: 235–292 (266.86 ± 34.41)	
Lamnidae				
*Isurus oxyrinchus*	2	M: 2	M: 178.5–182.5 (180.5 ± 43.97)	Specimens caught in gillnet and pelagic longline.
CARCHARHINIFORMES				
Triakidae				
*Hemitriakis indroyonoi*	2	F: 2	F: 100.6–105 (102.8 ± 3.11)	Two females landed in December 2017 and February 2018. Both specimens caught from Campbell Bay in Nicobar using pelagic longlines.
*Mustelus mosis*	7	F: 7	F: 85.2–108.5 (97.6 ± 8.60)	Three individuals landed together in March 2017 and four landed together in April 2018. All specimens caught using hook and line.
Hemigaleidae				
*Hemigaleus microstoma*	24	F: 7, M: 15, UK: 2	F: 65–109.9 (96.25 ± 17.19)	All specimens caught using trawl net, demersal longline and hook and line.
			M: 70.8–103.2 (94.42 ± 9.72)	
*Hemipristis elongata*	29	F: 10, M: 18, UK: 1	F: 93.1–211.1 (155.94 ± 42.17)	Four specimens caught using hook and line, four in demersal longline and seven in pelagic longlines.
			M: 95–183 (144.11 ± 22.77)	
*Paragaleus randalli*	169	F: 78, M: 91	F: 46–97.5 (86.44 ± 9.57)	Fourteen gravid females (n = 10 in April) recorded ranging from TL 87.5 to 97.5 cm, dissection of two gravid females revealed a litter size of two; three fully-developed embryos recorded ranging between TL 43.5 to 47.5 cm. Sex ratios of landings did not differ significantly from parity (F: M = 1: 1.16, χ^2^ [1, n = 169] = 1, p > 0.05). Size extension by 6.7 cm recorded.
			M: 43.5–102.5 (86.34 ± 8.75)	
Carcharhinidae				
*Carcharhinus albimarginatus*	295	F: 150, M: 137, UK: 8	F: 60.7–243.5 (103.41 ± 30.52)	One gravid female landed in February measuring TL 199.5 cm and 1 embryo of TL 21.5 cm landed in December, caught in pelagic longline; 25 recorded YOY ranging from TL 60.7 to 94 cm landed in March and April 2017, and Jan, Feb, April 2018. In April, more than 150 YOY of less than one meter landed by a pelagic longline—it was not possible to sample all these due to time constraints prior to the auction and only data from 24 specimens were recorded. Sex ratios of landings did not differ significantly from parity (F: M = 1: 0.91, χ^2^ [1, n = 287] = 0.588, p > 0.05).
			M: 21.5–249 (105.93 ± 30.49)	
*Carcharhinus altimus*	4	F: 1, M: 3	F: 90	Three YOY landed in March and April 2017 ranging from TL 90 to 128 cm. One specimen caught in pelagic longline and one in gillnet.
			M: 103–237.5 (156.16 ± 33.49)	
*Carcharhinus amblyrhynchoides*	7	F: 3, M: 2, UK: 2	F: 94–167.4 (138.46 ± 39.40)	One specimen caught in pelagic longline, gillnet and trawl net each.
			M: 194–206 (200 ± 56.93)	
*Carcharhinus amblyrhynchos*	1215	F: 652, M: 555, UK: 8	F: 51–217 (97.46 ± 30.73)	Four gravid females landed in January and February ranging between TL 157.5 and 186.5 cm. A total of 293 YOY ranging from TL 51 to 101 cm were recorded throughout the year. Significantly more females were landed than males (F: M = 1: 0.85, χ^2^ [1, n = 1207] = 7.79, p < 0.05).
			M: 55–206 (95.45 ± 27.44)	
*Carcharhinus amboinensis*	38	F: 21, M: 17	F: 134.5–295 (222.58 ± 35.98)	Five gravid females landed in February, July 2017, and April 2018, ranging from TL 217 to 295 cm. Size extension by 15 cm recorded.
			M: 138–233.2 (196.78 ± 36.08)	
*Carcharhinus brevipinna*	212	F: 95, M: 116, UK: 1	F: 59.7–284.5 (100.93 ± 32.5)	One gravid female of TL 206.5 cm and weighing 111 kg landed in April, caught in a gillnet; 49 YOY ranging from 59.7 to 84.7 TL cm landed in March and April. Sex ratios of landings did not differ significantly from parity (F: M = 1: 1.22, χ^2^ [1, n = 211] = 2.09, p > 0.05).
			M: 62.6–212 (94.07 ± 32.20)	
*Carcharhinus dussumieri*	80	F: 47, M: 33	F: 54.5–93.1 (82.79 ± 35.20)	Six gravid females ranging from TL 84.7 to 93.1 cm were landed between November 2017 and February 2018. Sex ratios of landings did not differ significantly from parity (F: M = 1: 0.70, χ^2^ [1, n = 80] = 2.45, p > 0.05).
			M: 22.3–92.5 (77.41 ± 35.45)	
*Carcharhinus falciformis*	71	F: 34, M: 34, UK: 3	F: 104–376.5 (181.69 ± 35.34)	One gravid female of TL 235.5 cm landed in February 2017, caught in gillnet. Sex ratios of landings shows parity (F: M = 1: 1, χ^2^ [1, n = 68] = 0 p > 0.05). Size extension by 26 cm recorded.
			M: 121–290.8 (180.33 ± 40.54)	
*Carcharhinus leucas*	32	F: 17, M: 14, UK: 1	F: 146–351 (265.61 ± 37.48)	Three gravid females of TL 309 to 351 cm landed in February and March 2018, in pelagic longlines and trawl nets.
			M: 124.5–274.8 (206.6 ± 35.71)	
*Carcharhinus limbatus*	108	F: 54, M: 53, UK: 1	F: 62.6–281 (112.6 ± 35.23)	Seventeen YOY landed in March and April 2018, with TL 61.5 to 77 cm. Sex ratios of landings did not differ significantly from parity (F: M = 1: 0.98, χ^2^ [1, n = 107] = 0.00934, p > 0.05). Size extension by 10 cm recorded.
			M: 61.5–231.8 (106.46 ± 35.34)	
*Carcharhinus longimanus*	19	F: 10, M: 9	F: 99.5–200 (137.89 ± 32.91)	One immature male landed in December of TL 96 cm. All specimens caught in pelagic longlines.
			M: 96–198.2 (141.53 ± 35.74)	
*Carcharhinus macloti*	5	F: 3, M: 2	F: 81.5–88.5 (85 ± 37.24)	Two specimens caught in gillnet, one in hook and line.
			M: 85–85.5 (85.25 ± 36.38)	
*Carcharhinus melanopterus*	30	F: 13, M: 20, UK: 2	F: 57.5–152.5 (84.88 ± 27.1)	One gravid female landed in March 2017 measuring TL 133 cm, caught in a gillnet; four male and two female YOY landed in May with TL 57.5 to 63 cm, six caught by hook and line.
			M: 57.5–129 (86.72 ± 28.98)	
*Carcharhinus plumbeus*	1	M: 1	M: 81	YOY, caught by hook and line.
*Carcharhinus sorrah*	48	F: 20, M: 26, UK: 2	F: 74–173.7 (118.37 ± 25.35)	Size extension by 3.5 cm recorded. Two specimens caught in trawl net, 17 in pelagic longline, seven in demersal longline, two in hook and line, nine in gillnet.
			M: 72.5–199.5 (116.45 ± 28.88)	
*Loxodon macrorhinus*	1549	F: 678, M: 852, UK: 19	F: 39.2–103.2 (84.24 ± 10.04)	Thirty-four gravid females landed between November 2017 to April 2018, with TL 85.5 to 98 cm; dissection of four gravid females revealed a litter size of two. Six fully-developed embryos pulled out from the gravid female measured TL 45.1 to 49.2 cm; 6 YOY of TL 32.6 to 54 cm, with open umbilical scars landed in March 2017 and 2018. Significantly more males than females were landed (F: M = 1: 1.25, χ^2^ [1, n = 1530] = 14.3771, p < 0.05). A size extension by 4.3 cm was recorded.
			M: 25–102 (84.46 ± 9.41)	
*Negaprion acutidens*	9	F: 5, M: 4	F: 66.7–265 (165.18 ± 92.62)	Three male YOY landed in March ranging from 63.4 to 70.6 TL. Three specimens caught in pelagic longline.
			M: 63.4–210 (76.2 ± 16.33)	
*Rhizoprionodon acutus*	102	F: 54, M: 47, UK: 1	F: 49.4–96.5 (77.69 ± 10.87)	Three gravid females landed ranging from TL 90 to 92.8 cm, one YOY of TL 61.5 cm, with a half healed umbilical scar landed in March 2018. Sex ratios of landings did not differ significantly from parity (F: M = 1: 0.87, χ^2^ [1, n = 101] = 0.485, p > 0.05).
			M: 66–93.7 (76.90 ± 7.53)	
*Triaenodon obesus*	14	F: 6, M: 8	F: 62–150.5 (91 ± 31.84)	One YOY with healing umbilical scar of TL 62 cm landed in December 2017. Two specimens were caught in gillnet, three in hook and line, one in demersal longline, two in pelagic longline.
			M: 91.5–136 (105.75 ± 14.09)	
Sphyrnidae				
*Sphyrna lewini*	421	F: 229, M: 177, UK: 14	F: 50–276 (100.57 ± 35.41)	Forty YOY ranging from TL of 61 to 86 cm were observed while no gravid females were recorded. Sex ratios of landings differed significantly from parity towards females (F: M = 1: 0.77, χ^2^ [1, n = 406] = 6.66, p < 0.05).
			M: 35.5–238 (106.18 ± 35.25)	
*Sphyrna mokarran*	2	F: 1, M: 1	F: 158.5, M: 64.5	Both specimens caught in trawl nets.

The table includes total number of individuals; sizes (total length (TL) for sharks) by sex (F—female; M—male; UK—unknown), presence of gravid females and young of year (YOY). Results of Chi-square tests of parity in sex ratios are provided for species with ≥ 50 individuals recorded, and fishing gears used to catch the species are provided where applicable.

### Use of fishing gears and fishing grounds

Twenty-one species were recorded interacting with gillnets, hook and line, and pelagic longlines, 18 species were recorded interacting with demersal longlines, 14 species with trawl nets and two species (*Centrophorus atromarginatus* (n = 1) and *C*. *granulosus* (n = 6)) with deep-sea longlines. Certain species were only recorded in one type of gear. For example, *Alopias pelagicus* (n = 28), *A*. *superciliosus* (n = 6), *C*. *longimanus* (n = 19) and *Hemitriakis indroyonoi* (n = 2) were only associated with pelagic longlines; *Mustelus mosis* (n = 7) were only recorded from hook and line; and *S*. *mokarran* (n = 2) were only recorded in trawl nets.

**Fig 2:**
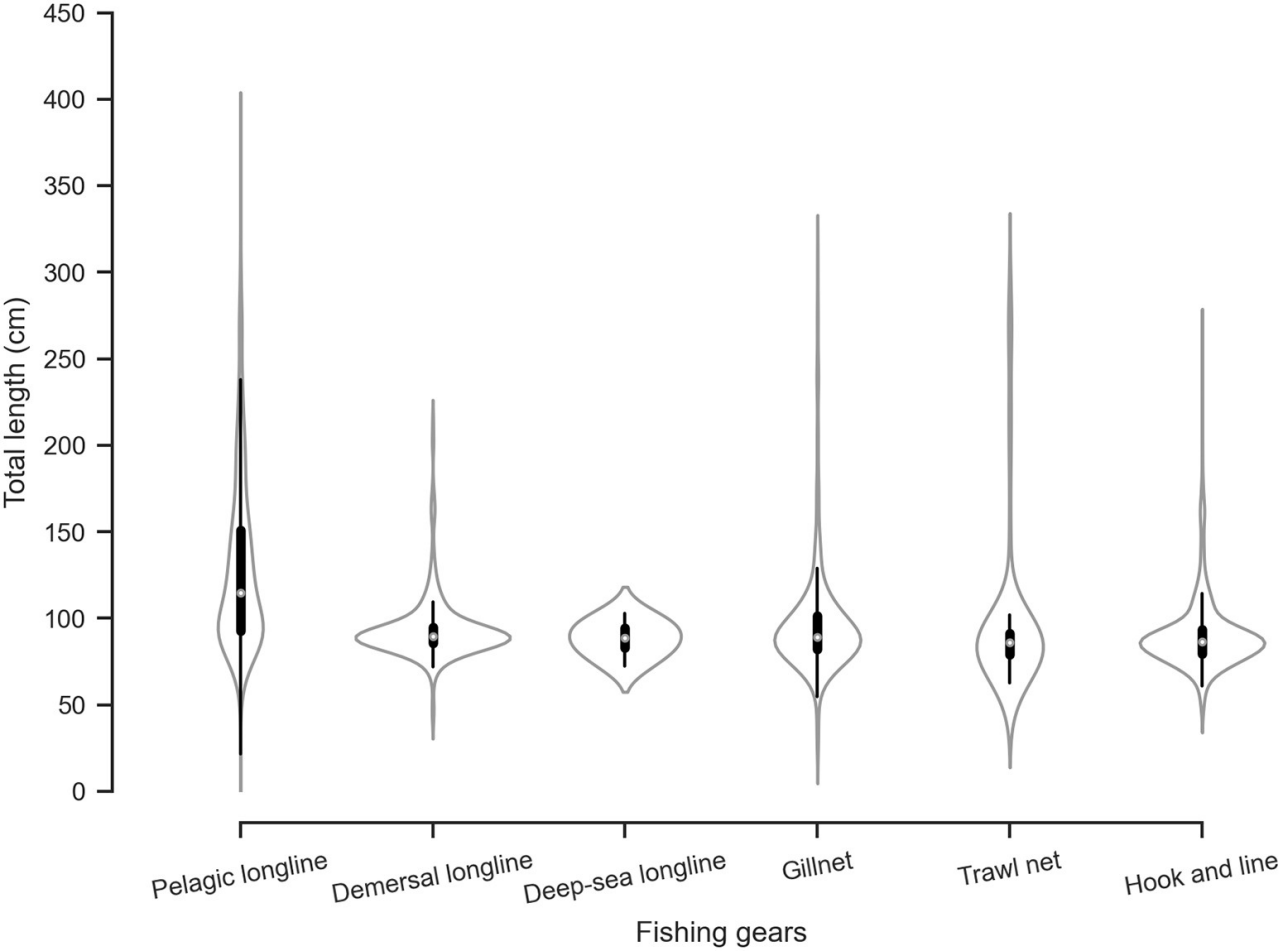
Total length (in cm) of sharks landed across the different fishing gear used on the islands. The white dot represents the median, the thick grey bar in the center represents the interquartile range, the thin gray line represents the data distribution except for the outliers, and the kernel represents the distribution of the data.

Further, there was a significant difference between the TL of sharks caught depending on the type of fishing gears used (*f* (5, 2,146) = 88.66, *p* < 0.005). Sharks landed by pelagic longliners had a wide TL range from 21.5 to 376.5 cm (mean of 124.90 ± 49.83); those in demersal longlines had a TL range from 42 to 214.5 cm (mean 18.81 ± 93.76); those in deep-sea longlines (>200 m) had a TL range from 72.5 to 103 cm (mean of 88.3 ± 10.80); those in gillnets had a TL range from 25 to 312.5 cm (mean of 97.49 ± 34.26); those in trawl nets had a TL range from 50 to 297.9 cm (mean of 47.67 ± 97.65); and those from hook and line had a TL range of 46 to 266.7 cm (mean of 47.67 ± 97.65) (Fig 2). The fishing grounds with frequency of each fishing gear used across the islands is provided in Fig 1.

### Seasonality, size frequency, and length-weight relationships

The following section provides details of the size frequency, seasonality and length-weight relationships of the six dominant shark species landed. Additional information on all species, including sex ratios where applicable and recorded extensions in maximum size for seven species, are provided in Table 1. For the non-dominant shark species in landings, of the 2,258 male individuals whose maturity was recorded, 35.93% of sharks were mature. The majority of specimens from small-bodied species (TL < 1.5 m) were mature (63.48%) whereas the majority of specimens from large-bodied species (TL > 1.5 m) were immature (84.79%).

#### Loxodon macrorhinus

**Fig 3:**
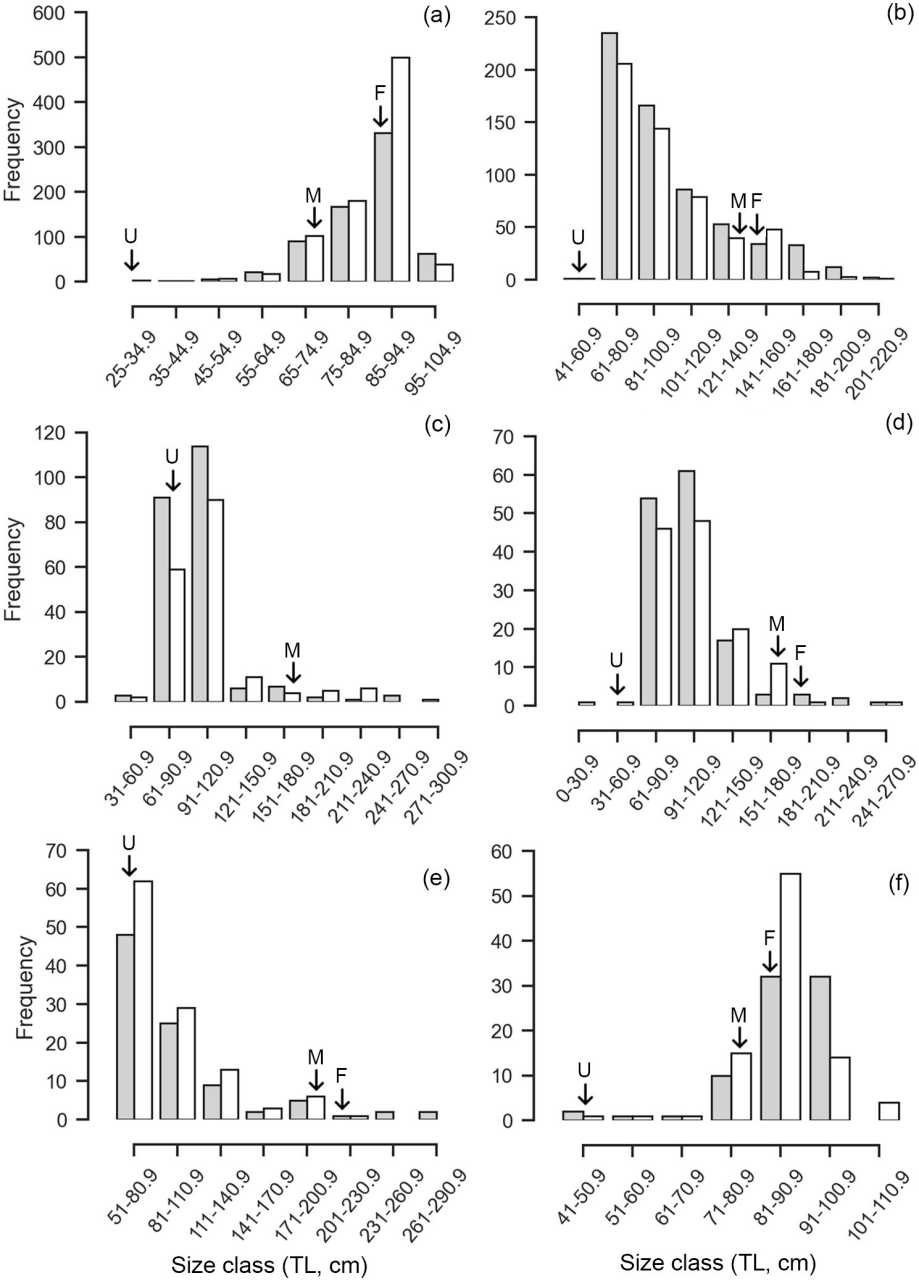
Size frequency distribution for males and females for the six most commonly landed shark species. (a) *L*. *macrorhinus*, (b) *C*. *amblyrhynchos*, (c) *S*. *lewini*, (d) *C*. *albimarginatus*, (e) *C*. *brevipinna*, and (f) *P*. *randalli*. The grey bars represent females and the white bars represent males. The arrows represent the smallest individual representing young of year with the presence of an umbilical scar ‘U’, ‘F’ the smallest gravid females recorded, and ‘M’ the smallest recorded mature males.

**Fig 4:**
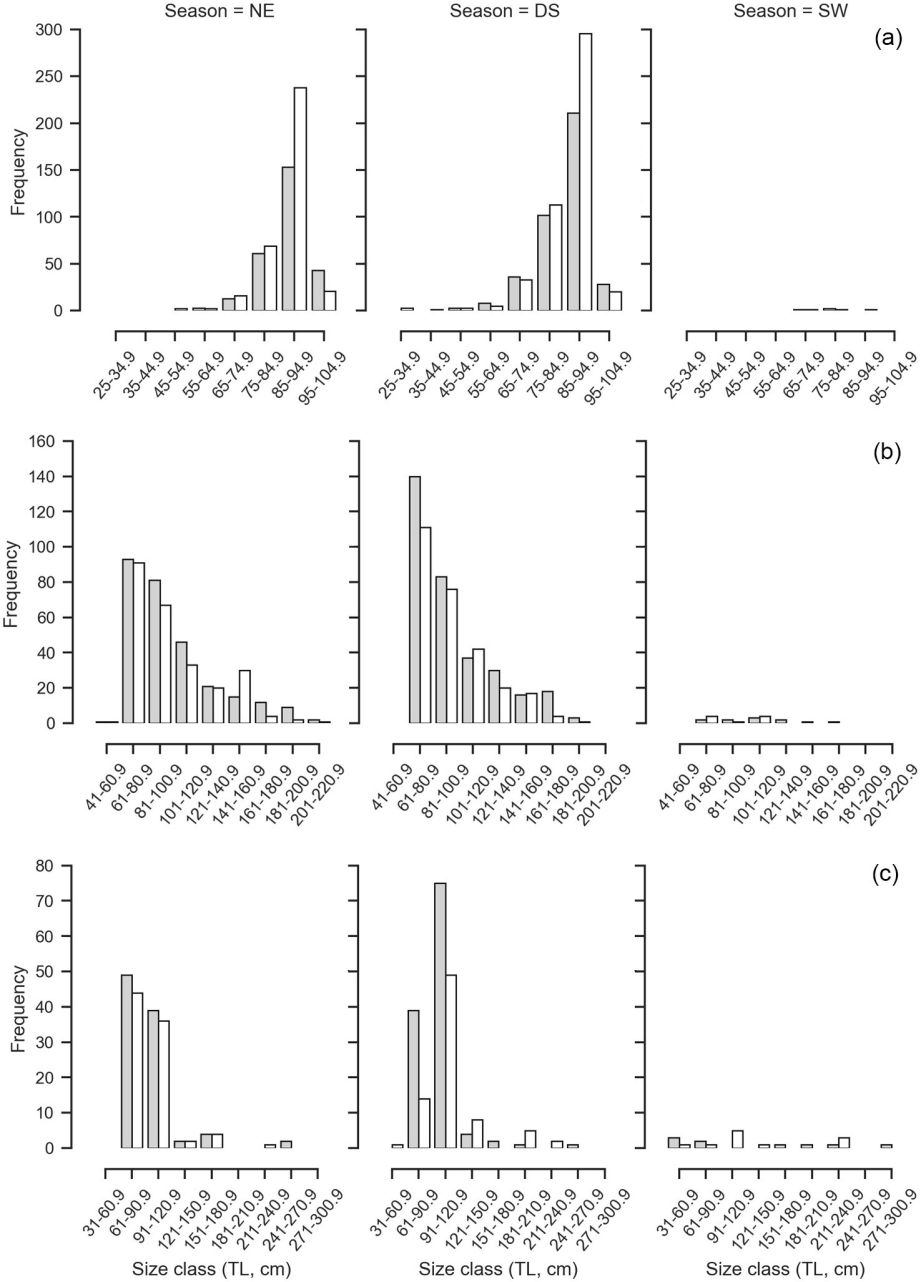
The seasonal size distribution of male and females for the most commonly landed shark species. (a) *L*. *macrorhinus*, (b) *C*. *amblyrhynchos*, (c) *S*. *lewini*. The seasons are north-east monsoon (NE) (October–January), inter-monsoon or dry season (DS) (February–May) and south-west monsoon (SW) (June–September). The grey bars represent females and the white bars represent males.

**Fig 5:**
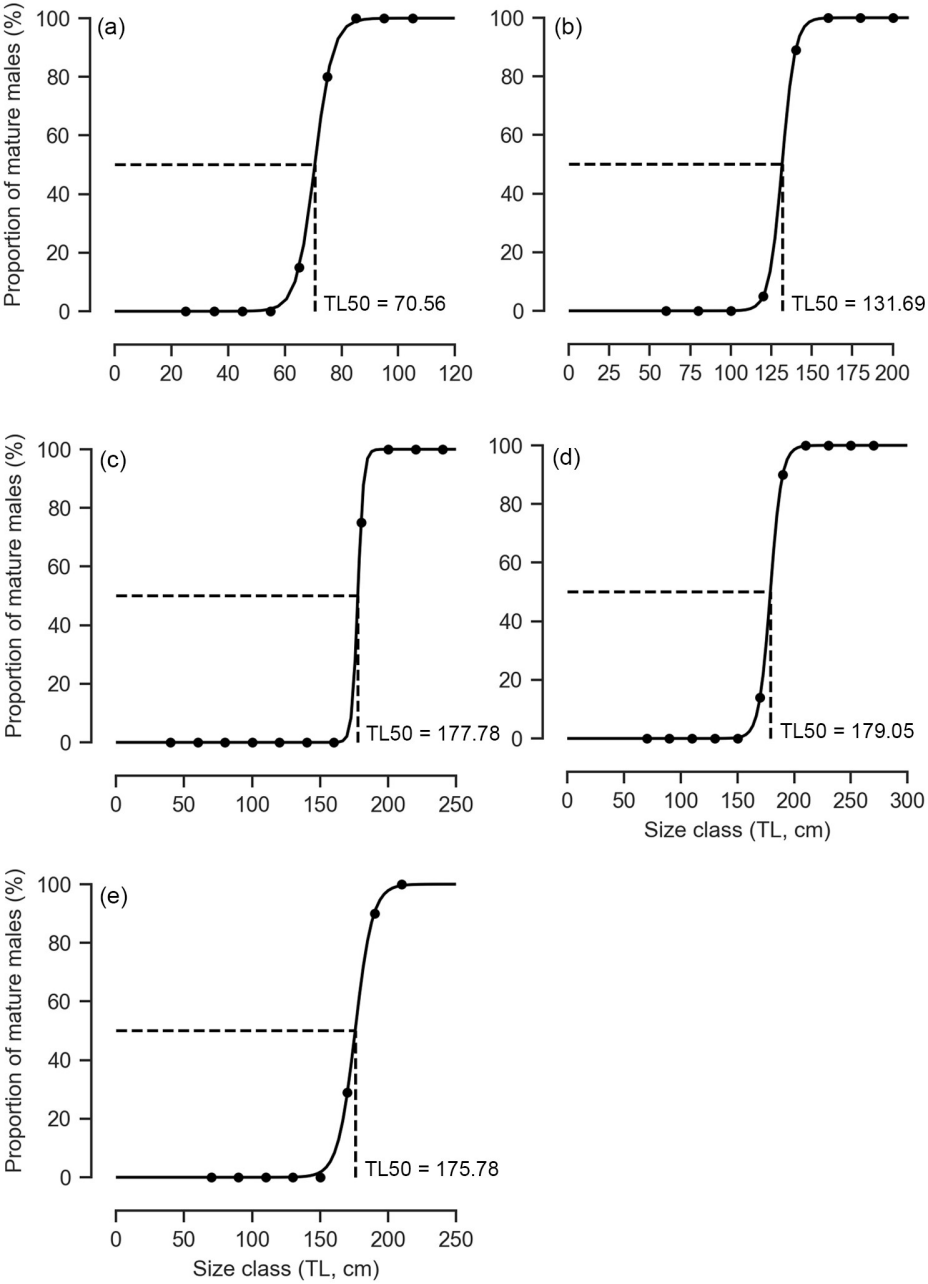
Percentage of mature males with total length (TL) for sharks at 50% maturity for the five most commonly landed shark species. (a) *L*. *macrorhinus*, (b) *C*. *amblyrhynchos*, (c) *S*. *lewini*, (d) *C*. *albimarginatus*), and (e) *C*. *brevipinna*.

**Fig 6:**
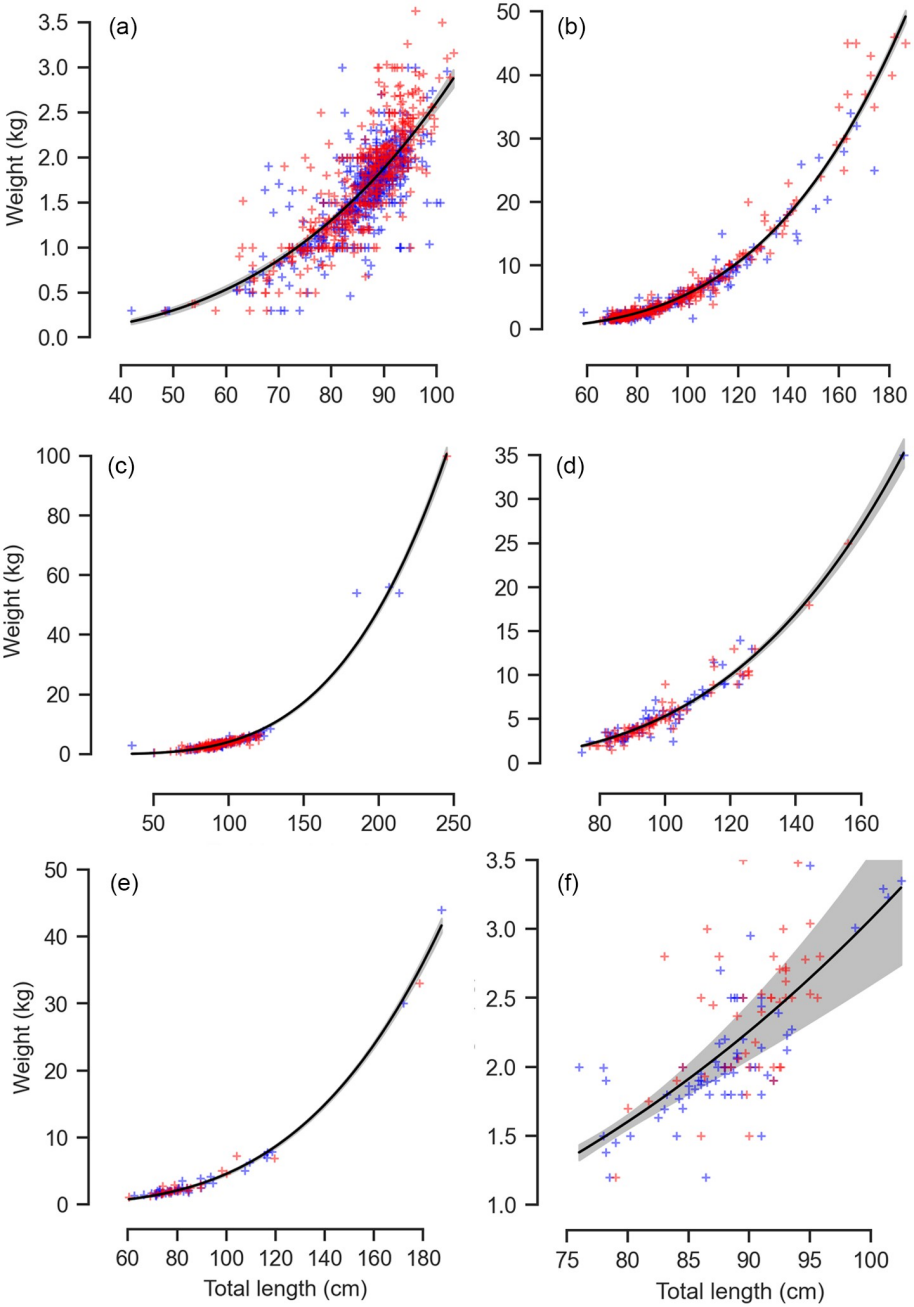
Length and weight relationships between total body mass (kg) and total length (cm) for the six most commonly landed shark species. (a) *L*. *macrorhinus*, (b) *C*. *amblyrhynchos*, (c) *S*. *lewini*, (d) *C*. *albimarginatus*, (e) *C*. *brevipinna*, and (f) *P*. *randalli*. The red marks represent females and the blue marks represent males.

The size frequency of *L*. *macrorhinus* followed a unimodal size distribution where mature individuals of TL 85–95 cm (n = 830, 54.35%) were dominantly landed across both sexes (Fig 3). Landings were variable across seasons with a peak during the dry season (n = 909) followed by NE monsoon (n = 632) and low landings during the SW monsoon (n = 8) (Fig 4). Of the 852 males, 75.94% were mature. The smallest immature male was 32.6 cm whereas the largest was 78.1 cm. The smallest mature male was 67.3 cm, whereas the largest was 102 cm with a TL_50_ of 70.56 cm (Fig 5, S1 Table). Landings of gravid females at various stages of embryo development were observed throughout the year, whereas YOY were observed in the months of March and April of 2017 and 2018, with one individual observed in January 2018. The length-weight relationships differed between sexes, where females showed positive allometry (b = 3.39), whereas males showed growth in a negative allometric manner (b = 2.68) in proportion with the cube of the length (Fig 6, S2 Table).

#### Carcharhinus amblyrhynchos

Immature individuals of size class TL 61–81 cm dominated landings across both sexes (n = 441, 38.28%), followed by size class 81–100.9 cm (n = 310, 26.90%) (Fig 3). Landings were variable across seasons with a peak during the dry season (n = 633) followed by NE monsoon (n = 559) and a lower number of individuals landed during the SW monsoon (n = 23) (Fig 4). Of the 555 males, 16.19% were mature. The smallest mature male was TL 126.3 cm whereas the largest was 206 cm. The TL_50_ of males was 131.69 cm (Fig 5, S1 Table). The length-weight relationships of both sexes showed a positive allometric relationship (female b = 3.52; male b = 3.28), in proportion with the cube of the length (Fig 6, S2 Table).

#### Sphyrna lewini

Landings of *S*. *lewini* were dominated by the size class TL 91 to 120.9 cm (n = 204, 50.37%), followed by size class 61–90.9 cm (n = 150, 37.03%) (Fig 3). Landings were variable across seasons with a peak during the dry season (n = 211) followed by NE monsoon (n = 189) whereas comparatively fewer landings were recorded during the SW monsoon (n = 21) (Fig 4). Of 177 males, 9.65% were mature. Immature individuals measured 35.5 to 170.4 cm TL, whereas mature individuals measured 177 to 238 cm TL with a TL_50_ of 177.78 cm (Fig 5, S1 Table). The length-weight relationships did not significantly differ between sexes, where both showed growth in a positive allometric manner (female b = 3.60; male b = 3.57), in proportion with the cube of the length (Fig 6, S2 Table).

#### Carcharhinus albimarginatus

**Fig 7:**
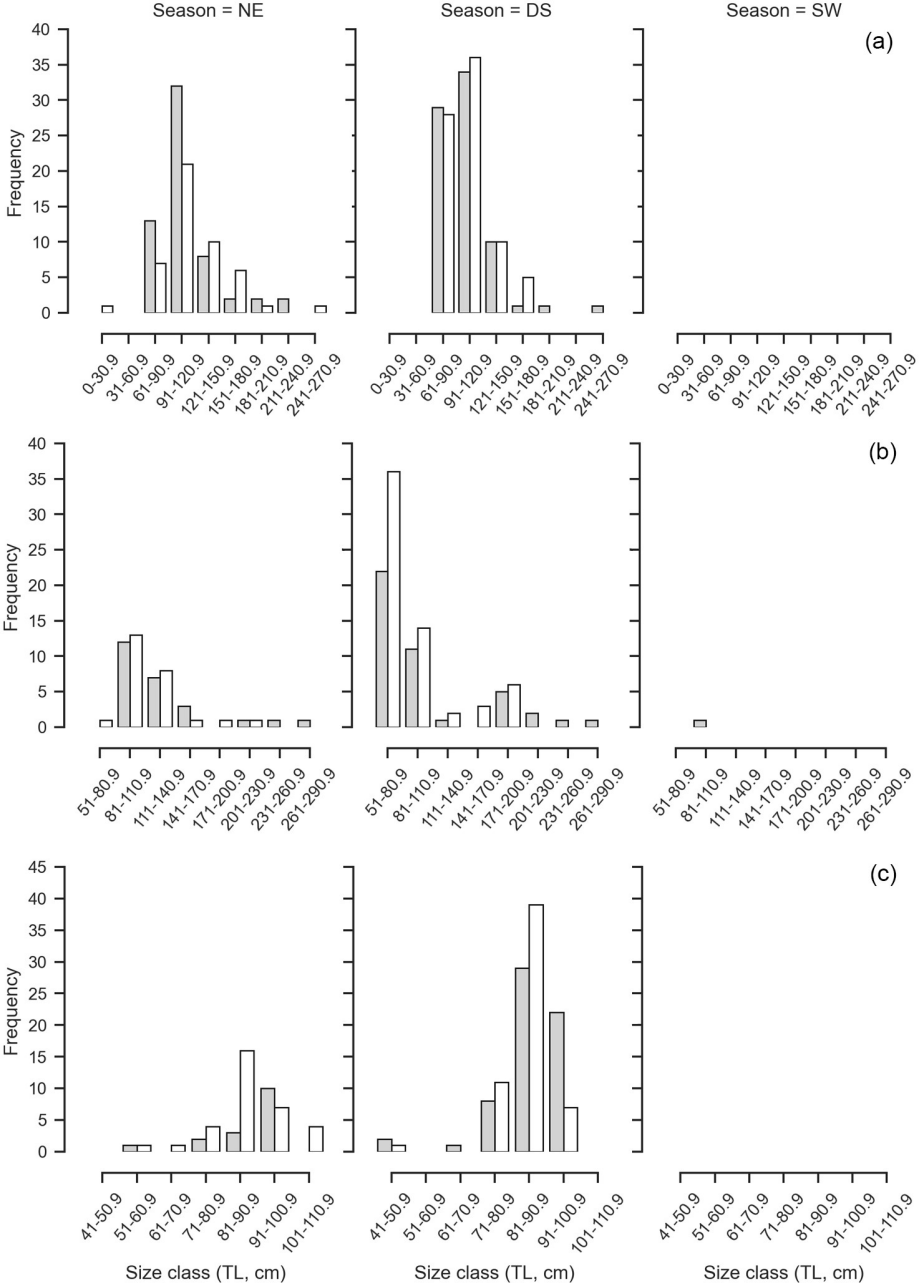
The seasonal size distribution of male and females for the most commonly landed shark species. (a) *C*. *albimarginatus*, (b) *C*. *brevipinna*, and (c) *P*. *randalli*. The seasons are north-east monsoon (NE) (October–January), inter-monsoon or dry season (DS) (February–May) and south-west monsoon (SW) (June–September). The grey bars represent females and the white bars represent males.

The size frequency of *C*. *albimarginatus* followed a unimodal size distribution where immature individuals of size class TL 91–121 cm dominated landings (n = 109, 40.37%) across both sexes (Fig 3). Landings were variable across seasons with a peak during the dry season (n = 177), followed by NE monsoon (n = 118) with none recorded during the SW monsoons (Fig 7). Of the 137 males, 4.47% were mature. The smallest mature male was 173 cm whereas the largest was 249 cm. The TL_50_ of males was 179.05 cm (Fig 5, S1 Table). The length-weight relationships showed that males and females did not differ significantly in their average weight for a given length, and weight increased in a positive allometric manner (female b = 3.45; male b = 3.43), in proportion with the cube of the length (Fig 6, S2 Table).

#### Carcharhinus brevipinna

Individuals of the size class TL 51–80.9 cm (n = 110, 52.88%) dominated landings, followed by size class 81–110.9 cm (n = 54, 25.96%) where male YOY (n = 62) were caught more than females (n = 48) (Fig 3). Landings were variable across seasons and differed in sex and size. Landings peaked during the dry season (n = 159) followed by NE monsoon (n = 52) with low landings during the SW monsoon (n = 1) (Fig 7). Of the 116 males sampled, 10.11% were mature. Mature males ranged from TL 172 to 212 cm, whereas immature males ranged from TL 62.6 to 175.78 cm. The TL_50_ of males was 175.78 cm (Fig 5, S1 Table). The length-weight relationships did not significantly differ between sexes, where both sexes showed growth in a positive allometric manner (female b = 3.36; male b = 3.59), in proportion with the cube of the length (Fig 6, S2 Table).

#### Paragaleus randalli

The size frequency followed a unimodal distribution where females of size classes TL 81–90.9 cm (n = 87, 51.47%) dominated landings, followed by 91–100.9 cm (n = 46, 27.2%) (Fig 3). Landings peaked during the dry season (n = 120) followed by a decrease in NE monsoon (n = 49) whereas no landings were observed during the SW monsoons (Fig 7). Of 91 males recorded, 93.4% were mature. The smallest immature individual measured 43.5 cm whereas the largest measured 76.5 cm. The smallest mature individual measured 74.5 cm, whereas the largest measured 106.2 cm. The length-weight relationships showed that males and females growth was in a negative allometric manner (female b = 2.46; male b = 2.96), in proportion with the cube of the length (Fig 6, S2 Table).

### Ray species composition

Species from the *Dasyatidae* family dominated landings, accounting for 11 of the 21 species, and 63.06% of the total landings. The three most common rays landed were *Pateobatis jenkinsii* (n = 241, 21.71%), *Himantura leopard*a (n = 206, 18.55%) and *H*. *tutul* (n = 95, 8.55%), representing 48.82% of the total ray landings. A species list and summary of biological data for rays is provided in Table 2.

**Table 2:** Summary of biological data for rays landed.

Species	n	n by sex of individuals	Size (DW cm)	Additional notes
RHINOPRISTIFORMES				
Rhinidae				
*Rhynchobatus* sp.	3	F: 1, M: 2		Head and fins were cut prior to landing so species-level identification was not possible.
Glaucostegidae				
*Glaucostegus typus*	14	F: 7, M: 7	F: 187.5–230.2 (210.64 ± 15.51)	All specimens caught in trawl nets.
			M: 198.2–235.5 (217.4 ± 11.90)	
MYLIOBATIFORMES				
Gymnuridae				
*Gymnura poecilura*	3	F: 3	F: 98–105.1 (90.57 ± 17.36)	Two specimens caught in trawl nets and one in demersal longline.
Dasyatidae				
*Himantura leoparda*	206	F: 83, M: 121, UK: 2	F: 42–136.5 (94.24 ± 25.19)	Sex ratios of landings differed significantly from parity towards males (F: M = 1: 1.45, χ^2^ [1, n = 204] = 7.07, p < 0.05). Caught in trawl nets, gillnet, hook and line and demersal longlines.
			M: 41.7–153.2 (96.84 ± 19.15)	
*Himantura tutul*	95	F: 55, M: 39, UK: 1	F: 26.5–145 (119.03 ± 24.39)	Sex ratios of landings did not differ significantly from parity (F: M = 1: 0.70, χ^2^ [1, n = 94] = 2.72, p > 0.05). Caught in trawl nets, gillnet, hook and line and demersal longlines.
			M: 65.5–138.4 (115.2 ± 13.30)	
*Himantura uarnak*	27	F: 15, M: 12	F: 56.6–128 (95.64 ± 23.06)	Caught in demersal longline, hook and line and trawl net.
			M: 72–123.3 (102.23 ± 15.45)	
*Himantura undulata*	2	F: 2	F: 108–119.1 (113.55 ± 7.84)	Specimens caught in demersal longlines.
*Maculabatis gerrardi*	13	F: 7, M: 6	F: 21–107 (82.52 ± 29.67)	Five specimens caught in trawl nets.
			M: 60–81 (72.03 ± 8.03)	
*Neotrygon* sp.	19	F: 7, M: 11, UK: 1	F: 39–52.5 (44.82 ± 4.02)	Individuals could not be identified to the species level and likely comprised of three species—*N*. *orientalis*, *N*. *caerulopunctata*, *N*. *indica*.
			M: 35–45.5 (39.05 ± 3.48)	
*Pastinachus* sp.	35	F: 11, M: 23, UK: 1	F: 73–126.2 (101.78 ± 19.39)	Individuals could not be identified to the species level and likely comprised of two species—*P*. *ater* and *P*. *gracilicaudus*.
			M: 60.8–228.5 (108.59 ± 31.88)	
*Pateobatis fai*	58	F: 31, M: 26, UK: 1	F: 62–152.5 (122.61 ± 20.16)	Sex ratios of landings did not differ significantly from parity (F: M = 1: 0.83, χ^2^ [1, n = 57] = 0.43, p > 0.05). Caught in trawl nets, gillnets, hook and line and demersal longline.
			M: 76.5–194 (122.06 ± 28.95)	
*Pateobatis jenkinsii*	241	F: 144, M: 96, UK: 1	F: 37.5–138.5 (98.96 ± 31.56)	Sex ratios of landings differed significantly from parity towards females (F: M = 1: 0.66, χ^2^ [1, n = 240] = 9.6, p < 0.05). Caught in trawl nets, gillnet, hook and line and demersal longlines.
			M: 64.3–122.4 (98.94 ± 31.55)	
*Taeniurops meyeni*	2	F: 1, M: 1	F: 108.5, M: 100.4	Landings in the month of December 2017 and January 2018. Both specimens caught in demersal longline.
*Urogymnus asperrimus*	2	F: 1, M: 1	F: 109, M: 97	Landings in the month of February and May 2018.
Myliobatidae				
*Aetomylaeus vespertilio*	2	F: 1, M: 1	F: 166, M: 100.4	Landings in the month of January and March 2018. Both specimens caught in trawl nets.
Aetobatidae				
*Aetobatus flagellum*	2	F: 2	F: 133.5–136.5 (135 ± 2.12)	Both specimens caught in demersal longlines.
*Aetobatus ocellatus*	38	F: 24, M: 13, UK: 1	F: 62.5–156.2 (122.03 ± 26.58)	Caught in gillnet, trawl net, hook and line, and demersal longline.
			M: 89–153.5 (118 ± 24.1)	
Rhinopteridae				
*Rhinoptera jayakari*	19	F: 9, M: 9, UK: 1	F: 75.2–100.5 (98.96 ± 31.60)	One gravid female landed in January measuring DW 96 cm. Two specimens were caught in trawl nets, one in gillnet, one in demersal longline.
			M: 84.4–96 (99.33 ± 34.19)	
Mobulidae				
*Mobula kuhlii*	8	F: 8	F: 59.5–125 (107.36 ± 20.56)	Two specimens caught in gillnets, two in hook and line.
*Mobula mobular*	3	F: 1, M: 2	F: 205	One specimen caught in demersal longline and one in gillnet.
			M: 205.5–213 (209.25 ± 5.30)	
*Mobula thurstoni*	13	F: 5, M: 7, UK: 1	F: 78–167 (131.32 ± 33.72)	One specimen caught in demersal longline, one in trawl net.
			M: 126.5–158.7 (149.31 ± 10.77)	
*Mobula* sp.	27	F: 13, M: 9, UK: 5	F: 113–270.5 (163.76 ± 51.04)	Individuals could not be identified to the species level due to improper photo documentation
			M: 112.3–218.3 (153.86 ± 38.07)	

The table includes total number of individuals; sizes (disc width (DW) for rays) by sex (F—female; M—male; UK—unknown), presence of gravid females and young of year (YOY). Results of Chi-square tests of parity in sex ratios are provided for species with ≥ 50 individuals recorded, and fishing gears used to catch the species are provided where applicable.

Three species of rays, *Aetobatus flagellum*, *H*. *tutul*, and *P*. *fai*, were recorded for the first time from the Andaman and Nicobar Islands, with *H*. *tutul* being a new record from India (Table 2, S1 Fig). Five species previously reported as possibly occurring on the islands by Kumar et al. [29] were confirmed: *Aetomylaeus vespertilio*, *Glaucostegus typus*, *H*. *undulata*, *Mobula kuhlii*, and *P*. *jenkinsii*.

Sixteen species of rays were recorded interacting with demersal longlines, 14 species with trawl nets, ten with gill nets, seven with hook and line, and two with pelagic longlines. No rays were recorded from deep-sea longlines. Certain species were caught exclusively in certain gears. For example, *A*. *vespertilio* (n = 2), and *Maculabatis gerrardi* (n = 13) were only caught with trawl nets.

Of the 513 male individuals recorded, 80.71% were mature. Sex ratios were calculated for four rays, *H*. *tutul*, *H*. *leoparda*, *P*. *fai* and *P*. *jenkinsii* (Table 2).

## Discussion

The fish landing surveys carried out for sharks and rays in the Andaman and Nicobar Islands have greatly contributed to the current knowledge of species diversity and biology for the south and south-east Asian region. Three ray species are new records for the Andaman and Nicobar Islands, including one new record for India, increasing the elasmobranch diversity for the Andaman and Nicobar Islands from 103 to 106, and for India to 152 [29]. With this continuing increase in species records, it is clear that additional efforts are required to fully document the diversity of sharks and rays in India. This high number of species recorded around the islands reflects the diverse habitats they support, but also their overlapping distribution with important fishing zones.

Only two species of deep-sea sharks (*Centrophorus* sp.) were recorded in this study despite recent additions of seven new records from the region [14, 28–30]. This was due to the logistical difficulties in sampling the large quantities of deep-sea sharks landed, along with time constraints between landings and transport to the storage units (S2 Fig). Currently, there is an ongoing targeted deep-sea shark fishery in the Andaman Islands that supplies the demand for shark liver oil [20]. Deep-sea sharks have rates of population increase that are on average less than half those of shelf and pelagic species and are some of the lowest levels recorded to date [42]. Population recovery rates also decrease with increasing depth, suggesting that these species are most susceptible to overexploitation [42]. These life-history traits do not allow them to sustain intense fishing pressure which can lead to rapid population declines. This has been previously documented in the Indian Ocean region with the collapse of deep-sea fisheries along the west coast of India and the Maldives occurring within a short time period after the beginning of their exploitation [22, 43]. Thus, we emphasise the urgency and importance of assessing the status and monitoring the populations of deep-sea sharks, as well as determining the socio-economic benefits and impacts of the trade in shark liver oil, so that management measures such as catch limits, gear restrictions, and spatial or temporal regulations can be put in place in order to avoid the collapse of this fishery.

Many rays (e.g., *Neotrygon* sp., *Pastinachus* sp.) could not be identified to the species level due to their tails being cut, difficulty in manipulation due to their weight, or traders transporting them before photo-documentation was possible. Ongoing taxonomic uncertainty for many ray species currently exists in India, where there is ambiguity in several species complexes. In order to address and resolve this, a robust taxonomic framework needs to be developed which can be used to better understand diversity and potential impacts from fishing pressure on key species. In the future, a combination of molecular techniques and long-term fishery-independent surveys need to be established to gain a holistic picture of diversity, as well as population trends, in the region.

Further, we identified *M*. *mosis* through visual morphological characteristics. However, the species has been identified as the Bengal smoothhound *Mustelus cf*. *mosis* in Thai waters which closely resembles the Arabian smoothhound *M*. *mosis*, but differences in the sequences of the NADH2 marker suggest it may not be a conspecific [44]. Thus, additional molecular and morphometric work is required to determine the true range of *M*. *mosis*, and whether it might be a different species in the Andaman Sea. Similarly, *P*. *longicaudatus* could be a senior synonym of *P*. *randalli* [45, 46], although due to the unavailability of the type specimen, the validity of this species has not been confirmed.

At many sites sampled around the world, smaller-sized species are predominantly landed, as many of the larger-bodied shark species have been overfished [47–50]. Similarly, on peninsular India, shark stocks have declined over the past decade with smaller, faster-growing shark species displacing larger, slower-growing species [5, 11, 51–54]. A decrease in the diversity of species landed has also been documented in areas with high fishing pressure. Indeed, Thailand, closer to Andaman and Nicobar Islands than to mainland India, has recorded a decrease in landings of larger sharks from 41 species in 2004 to 15 species in 2014–2015 [44]. However, our results indicated that this is not yet the case in the Andaman and Nicobar Islands as four of the six dominantly landed sharks are larger bodied shark species. This suggests that we are still at a point where informed management decisions can lead to the conservation of these populations. However, as gravid females, immature individuals and YOYs are being fished, the productivity, resilience and sustainability of these populations may have already been reduced [55].

The largest size range in sharks was recorded in landings from pelagic longlines and gillnets. While gillnets fish up to six nautical miles from the coast across the Andaman Islands, pelagic longlines fish exclusively beyond six nautical miles from the coast and within 12 nautical miles and are known to fish in waters from South Andaman to Nicobar. The high range of TL and non-specificity of gear catch could be ascribed to the gear size, fishing grounds, or inter-specific and intra-specific variations in activity patterns. In future, size-selectivity studies in relation to the catch by gear need to be conducted in order to determine gear modifications best suited for the susceptible life-history stages of threatened shark and ray populations.

This study emphasizes the overlap between critical habitats and fishing grounds as all life-stages for most species were recorded, highlighting their susceptibility to fishing pressure. Gravid females of 12 species were reported, with fishers confirming that they were fished in the waters of the Andaman and Nicobar Islands. Immature individuals of large shark species are being fished intensely, such as *C*. *albimarginatus*, *C*. *amblyrhynchos*, *C*. *brevipinna* and *S*. *lewini*, which is a reason for concern as these species exhibit particularly low productivity and growth rates leading to high susceptibility to anthropogenic pressure and are slow to recover from overexploitation [56]. The large quantities of YOY landed for these species suggests that these species might be using the islands as pupping or nursery grounds. *Carcharhinus brevipinna* and *S*. *lewini* have been recorded using inshore nursery areas for their young [57–60]. Thus, we recommend that these breeding and nursery grounds need to be identified and evaluated, and potentially temporally and spatially managed.

Sex ratios in landings differed across species and fishing gears, which could be due to confounding factors such as gear selectivity, fishing grounds, season, productivity, currents and bathymetry [61]. Significantly more females than males for *C*. *amblyrhynchos*, *S*. *lewini*, and *P*. *jenkinsii* suggests that females of these species dominate the populations in these waters. These are also aggregating species often exhibiting some degree of site fidelity [62–66], another ecological character that needs to be considered in spatial management. Similarly, for *L*. *macrorhinus*, and *H*. *leoparda*, significantly more males were landed than females, whereas parity was recorded for *C*. *falciformis*. In future, region-specific studies need to be carried out to assess sex-mediated spatial ecology for sharks and rays. Systematic sampling from fishing vessels across seasons would also be required to get fine-scale overlap between temporal and spatial distribution of shark and rays, as well as fishing gear specificity.

Landings for sharks peaked from November to April, coinciding with pelagic longlines targeting sharks during this time. Seasonal differences during the year could be ascribed to various factors such as the weather, access to fishing grounds, fishing gears used, and the ecology of the species fished. During the SW monsoon (May to September), the absence of landings at the Junglighat site could be due to the weather which makes it risky for fishers to go out fishing but also coincides with the seasonal ban on trawlers and pelagic longliners (April 15^th^ to May 31^st^).

It is noteworthy to highlight species diversity, quantities landed, and TL ranges were highest in pelagic longlines. Pelagic longline fisheries are considered to be the greatest threat to pelagic shark species [67] and contribute to the largest part of the global shark and ray catches [68]. Landings from these gears included threatened species such as *Alopias pelagicus*, *A*. *superciliosus*, *C*. *falciformis*, *C*. *longimanus*, and *S*. *lewini* which are migratory species. These species are listed under Appendix I (*C*. *longimanus*) and/or II of the Convention on the Conservation of Migratory Species of Wild Animals and Appendix II of the Convention on International Trade in Endangered Species of Wild Fauna and Flora, to ensure international cooperation for the conservation of these migratory species and to regulate their trade respectively. Convention on International Trade in Endangered Species of Wild Fauna and Flora specifically requires the development of a Non-Detriment Finding to assure that trade is not adversely impacting populations [69]. Since India is a signatory to these conventions, there is an urgent need for regional cooperation on many migratory species, as well as the development and implementation of Non-Detriment Findings to ensure the international trade in the listed species is sustainable, legal, and traceable. However, due to the increasing domestic market of sharks and rays for meat, regulation of the export restrictions alone will not address the sustainable harvest of sharks and rays, and thus there is an urgent need to update domestic regulations that can complement existing international approaches.

Given India’s long coastline of nearly 7,516 km, along with the multi-stakeholder and multi-gear nature of fisheries, it is challenging to comprehensively monitor landings of sharks and rays. While the Central Marine Fisheries Research Institute in India has the most comprehensive fisheries database dating back to 1947, it is restricted to peninsular India, with no data from the Andaman and Nicobar Islands. Here, the monitoring is undertaken by the Andaman and Nicobar Islands Directorate of Fisheries who broadly focuses on commercial fish stocks and does not include species-specific categories for sharks and rays [20]. Additionally, the Zoological Society of India, Fisheries Survey of India and Central Island Agricultural Research Institute conduct opportunistic surveys to document species diversity. We conducted this study in the Andaman Islands to fill this gap. However, additional studies are required to address ongoing taxonomic ambiguities, improve knowledge of species by expanding fisheries-independent monitoring, and to facilitate long-term species-specific monitoring to inform management and conservation measures.

Shark and ray species protected under the WLPA were rarely landed (only two individuals of *U*. *asperrimus* were recorded). Most of the species listed in the WLPA are found in estuarine habitats and are not likely to occur around the islands, including *A*. *cuspidata*, *G*. *gangeticus*, and *G*. *glyphis*. *R*. *djiddensis* listed in the WLPA does not appear to occur in India and the species complex could include *R*. *australiae* and *R*. *laevis* [70]. However, the latter two species are not protected under the WLPA. Similarly, *H*. *fluviatilis* could refer to *Pastinachus* sp. or *Urogymnus polylepis* [71, 72].

Anecdotal reports from fishers state that a few of these species (e.g., *Pristis* sp.) have not been seen or landed for over a decade (Z. Tyabji unpubl. data). This highlights the urgent need for amending the WLPA and to include Critically Endangered and Endangered species that occur in India to the list of protected species. However, species-selective bans in non-selective multi-gear fishery are difficult to implement, thus an amendment of the WLPA has to be combined with stakeholder engagement and other regulations such as catch quotas, fishing gear modifications, and spatial closures.

While there exists a 45-day shark fishing ban, there are no regulations for ray fishing, despite them being predominantly threatened species. Rays are extremely susceptible to overexploitation, with wedgefishes and giant guitarfishes being the most imperiled marine taxa globally [1, 70]. Susceptibility studies on the various shark and ray species in Papua New Guinea deemed *P*. *jenkinsii* at the highest risk in trawl fisheries [73]. This was one of the most dominant species landed in the Andaman and Nicobar Islands. This is concerning as most ray species utilize coastal areas which overlap with the majority of fisheries. Additionally, there is a developing targeted ray fishery in the islands (Z. Tyabji unpubl. data) due to the local demand for their meat and trade in their skins. Studies regarding the local population status and exploitation rate of rays on the islands are urgently required, followed by a prioritizing exercise which takes into account life-history traits, susceptibility to fishing pressures, and population recovery rate. Based on this, ray species that are most susceptible to overexploitation need to be identified and a management plan needs to be developed and implemented.

A combination of policy changes such as the identification and protection of critical shark and ray habitats and populations, setting catch quotas, introducing gear modifications, and implementing seasonal and temporal bans, are daunting tasks, but are required for mitigating over-exploitation, achieving conservation goals and maintaining the fisheries for social-economic well-being of the fishers. The collection of robust data on which to base these strategies is an immediate management action to be put in place. We recommend additional studies and continued long-term monitoring with a focus on threatened species in order to establish appropriate management measures. We also need to understand the socio-economic importance of shark and ray fisheries for the range of stakeholders and communities on the islands and the role of these fisheries in the supply chain of both domestic and global markets while designing management strategies. It is essential that policy formulation and changes are carried out with the involvement of fishers and local stakeholders for effective implementation.

## Supporting information

S1 Table Maturity size ranges, TL_50_ and r value for males of the five commonly landed shark species. r is a constant that increases in value with the steepness of the maturation schedule.
(DOCX)

S2 Table Maximum likelihood estimates of length and weight regression parameters for the six commonly landed shark species. (DOCX)

S1 Fig 1) *Aetobatus flagellum* (a) dorsal view (b) ventral view of the mouth; 2) Two colourations of *Himantura tutul* (a) dorsal view (c) denticles on the nuchal area (b) dorsal view (d) denticles on the nuchal area; 3) *Pateobatis fai* (a) dorsal view (b) ventral view (c) tail. (TIF)

S2 Fig Sharks and rays landed at the fish landing sites. Clockwise from top left: Deep-sea sharks caught from deep-sea longline landed at Burmanallah; Fishers take out sharks from the pelagic longline boats at Junglighat; Shark fins kept to dry; Landed rays are weighed, following which they will be transported to the storage units; Mature and immature sharks of various species landed at Junglighat.
(TIF)
