# Supplementary material for: Catch composition and life history characteristics of sharks and rays (Elasmobranchii) landed in the Andaman and Nicobar Islands, India
Source: PLoS One. 2020 Oct 29;15(10):e0231069. doi: 10.1371/journal.pone.0231069 (PMC7595311; doi:10.1371/journal.pone.0231069)
Supplement: S1 Table — r is a constant that increases in value with the steepness of the maturation schedule. (DOCX) [file pone.0231069.s001.docx]

**S1 Table. Maturity size ranges, TL_50_ and r value for males of the five commonly landed shark species.** ‘r’ is a constant that increases in value with the steepness of the maturation schedule.

| **Species** | **n** | **Size range (cm)** | **Size range at maturity (cm)** | **r** | **Lm50 (cm)** |
| --- | --- | --- | --- | --- | --- |
| *Loxodon macrorhinus* | 820 | 25 - 102 | 67.3 - 102 | 0.31 | 70.56 |
| *Carcharhinus amblyrhynchos* | 518 | 58.6 - 206 | 126.3 - 206 | 0.25 | 131.69 |
| *Sphyrna lewini* | 176 | 35.5 - 238 | 177 - 238 | 0.48 | 177.78 |
| *Carcharhinus albimarginatus* | 127 | 66.8 - 249 | 173 - 249 | 0.20 | 179.05 |
| *Carcharhinus brevipinna* | 87 | 62.6 - 212 | 172 - 212 | 0.15 | 175.78 |
