# Supplementary material for: Catch composition and life history characteristics of sharks and rays (Elasmobranchii) landed in the Andaman and Nicobar Islands, India
Source: PLoS One. 2020 Oct 29;15(10):e0231069. doi: 10.1371/journal.pone.0231069 (PMC7595311; doi:10.1371/journal.pone.0231069)
Supplement: S2 Table — (DOCX) [file pone.0231069.s002.docx]

**S2 Table. Maximum likelihood estimates of length and weight regression parameters for the six commonly landed shark species.**

| **Species** | **Sex** | **n** | **TL range (cm)** | **Weight range (kg)** | **a** | **b** | **SE** |
| --- | --- | --- | --- | --- | --- | --- | --- |
| *Loxodon macrorhinus* | Combined | 931 | 42 - 103.2 | 0.29 - 3.63 | 1.558E-06 | 3.111 | 0.097 |
|  | Male | 498 | 42 - 102 | 0.3 - 3 | 1.010E-05 | 2.687 | 0.136 |
|  | Female | 433 | 48.5 - 103.2 | 0.29 - 3.63 | 4.565E-07 | 3.392 | 0.134 |
| *Carcharhinus amblyrhynchos* | Combined | 590 | 58.6 - 186.5 | 1.21 - 46 | 5.554E-07 | 3.500 | 0.030 |
|  | Male | 277 | 58.6 - 174 | 1.21 - 34 | 1.473E-06 | 3.286 | 0.046 |
|  | Female | 313 | 65 - 186.5 | 1.3 - 46 | 5.086E-07 | 3.524 | 0.040 |
| *Sphyrna lewini* | Combined | 240 | 35.5 - 245 | 0.47 - 100 | 2.767E-07 | 3.582 | 0.031 |
|  | Male | 96 | 35.5 - 213.5 | 0.47 - 56 | 2.882E-07 | 3.577 | 0.080 |
|  | Female | 144 | 50.5 - 245 | 0.54 - 100 | 2.468E-07 | 3.602 | 0.019 |
| *Carcharhinus albimarginatus* | Combined | 144 | 74.5 - 173 | 1.2 - 35 | 6.765E-07 | 3.447 | 0.054 |
|  | Male | 65 | 74.5 - 173 | 1.2 - 35 | 7.202E-07 | 3.436 | 0.072 |
|  | Female | 79 | 77 - 156 | 1.5 - 25 | 6.515E-07 | 3.454 | 0.087 |
| *Carcharhinus brevipinna* | Combined | 75 | 60.4 - 187.5 | 1.11 - 44 | 4.109E-07 | 3.522 | 0.041 |
|  | Male | 46 | 62.6 - 187.5 | 1.25 - 44 | 2.959E-06 | 3.590 | 0.042 |
|  | Female | 29 | 60.4 - 178.5 | 1.11 - 33 | 8.555E-06 | 3.368 | 0.065 |
| *Paragaleus randalli* | Combined | 115 | 76 - 102.5 | 1.2 - 3.5 | 4.524E-06 | 2.915 | 0.289 |
|  | Male | 68 | 76 - 102.5 | 1.2 - 3.46 | 3.469E-06 | 2.968 | 0.287 |
|  | Female | 47 | 79 - 95.8 | 1.2 - 3.5 | 3.593E-05 | 2.464 | 0.658 |
