# Supplementary figures and images for: Catch composition and life history characteristics of sharks and rays (Elasmobranchii) landed in the Andaman and Nicobar Islands, India
Source: PLoS One. 2020 Oct 29;15(10):e0231069. doi: 10.1371/journal.pone.0231069 (PMC7595311; doi:10.1371/journal.pone.0231069)

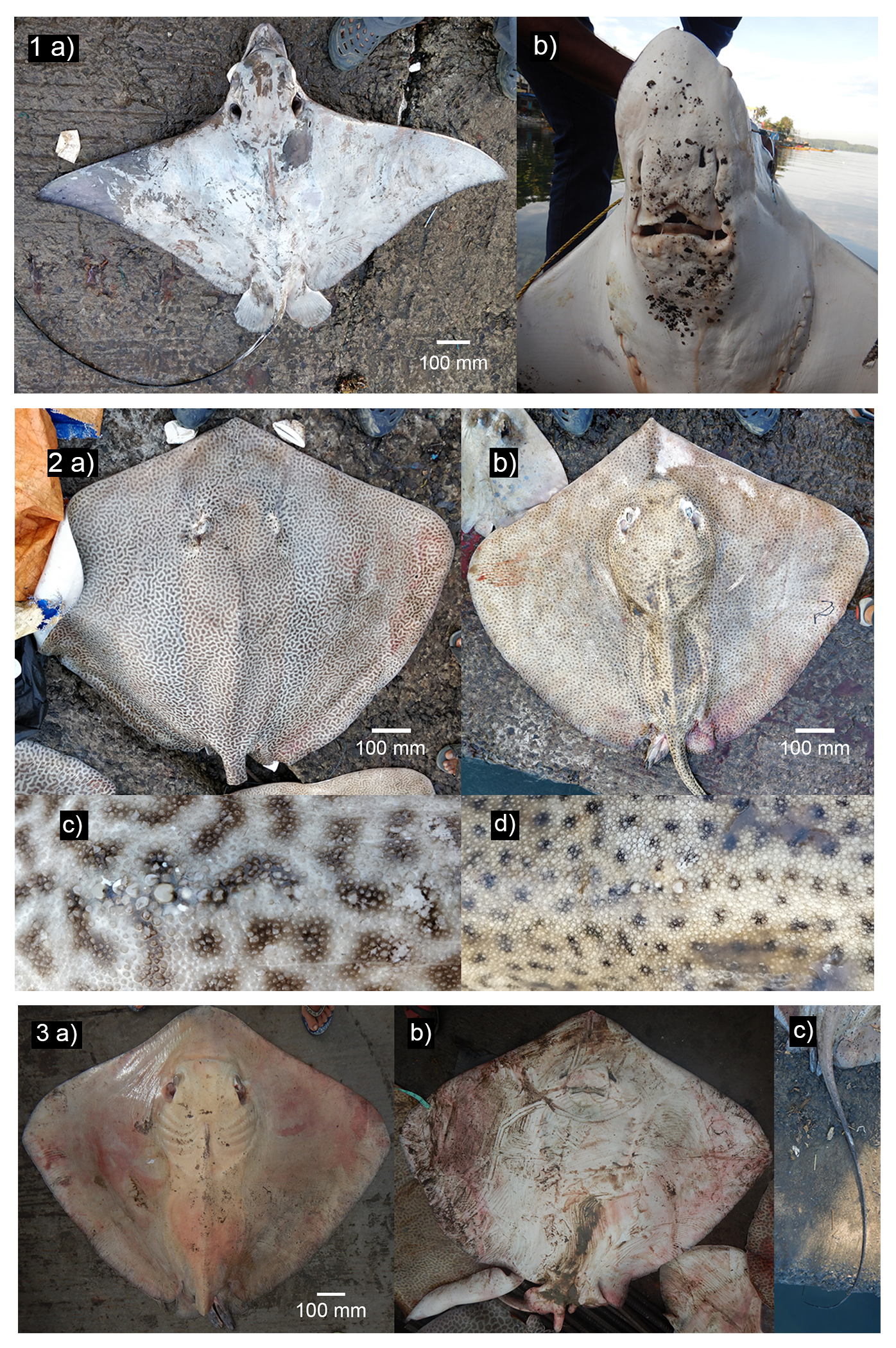

Supplement: S1 Fig — (TIF) [file pone.0231069.s003.tif]

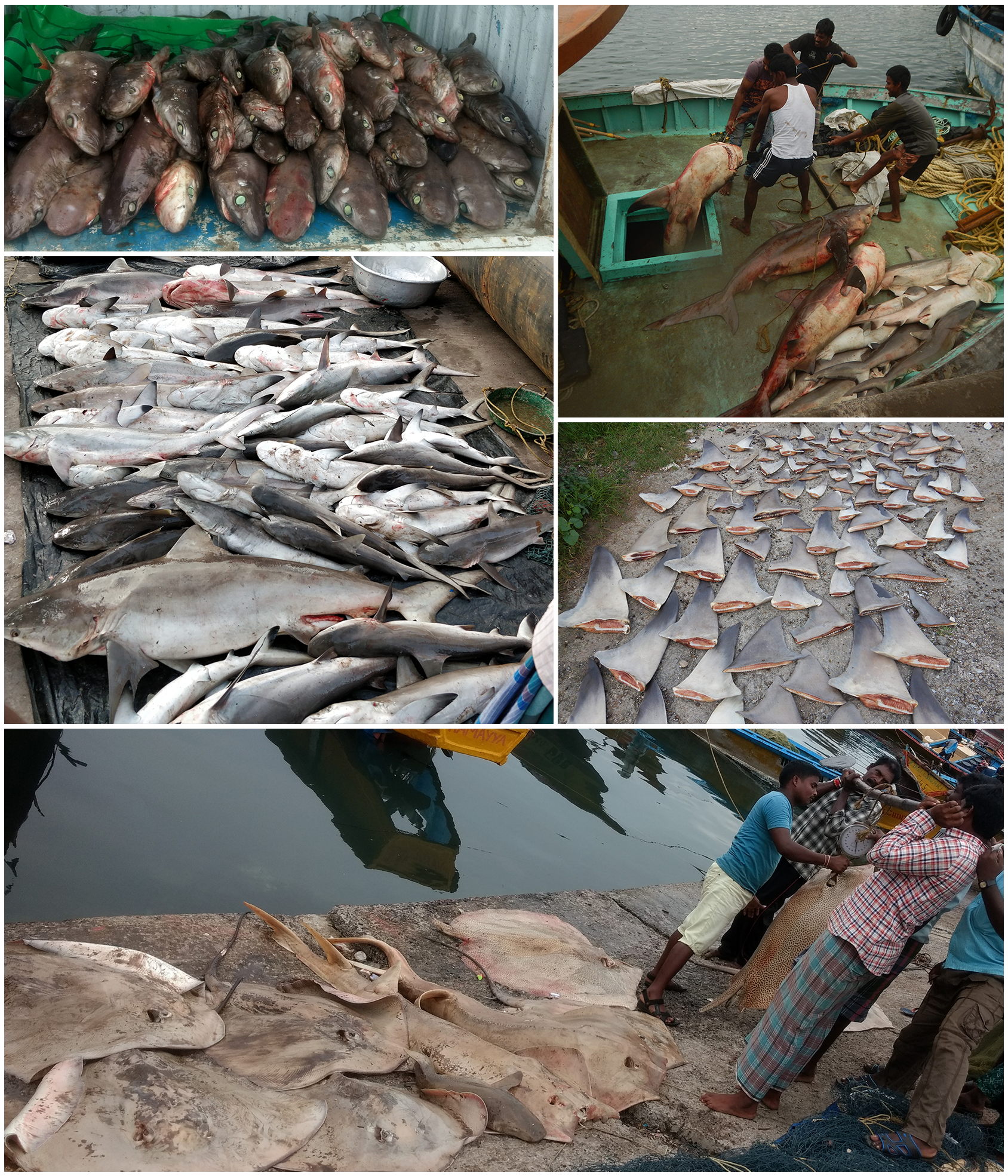

Supplement: S2 Fig — Clockwise from top left: Deep-sea sharks caught from deep-sea longline landed at Burmanallah; Fishers take out sharks from the pelagic longline boats at Junglighat; Shark fins kept to dry; Landed rays are weighed, following which they will be transported to the storage units; Mature and immature sharks of various species landed at Junglighat. (TIF) [file pone.0231069.s004.tif]
